# Green Synthesis of Near-Infrared Plasmonic Gold Nanostructures by Pomegranate Extract and Their Supramolecular Assembling with Chemo- and Photo-Therapeutics

**DOI:** 10.3390/nano12244476

**Published:** 2022-12-17

**Authors:** Mimimorena Seggio, Francesca Laneri, Adriana C. E. Graziano, Marta Maria Natile, Aurore Fraix, Salvatore Sortino

**Affiliations:** 1 PhotoChemLab, Department of Drug and Health Sciences, University of Catania, 95124 Catania, Italy; 2 ICMATE-CNR Institute of Condensed Matter Chemistry and Technologies for Energy, National Research Council, Department of Chemical Science, University of Padova, 35131 Padova, Italy

**Keywords:** green synthesis, light, supramolecular hybrid nanostructures, cyclodextrin polymers, photothermia

## Abstract

Au nanostructures exhibiting a localized surface plasmon resonance in the near-infrared spectral window are obtained in a single, green step at room temperature by pomegranate extract in the presence of a highly biocompatible β-cyclodextrin branched polymer, without the need of preformed seeds, external reducing and sacrificial agents, and conventional surfactants. The polymeric component makes the Au nanostructures dispersible in water, stable for weeks and permits their supramolecular assembling with the chemotherapeutic sorafenib and a nitric oxide (NO) photodonor (NOPD), chosen as representative for chemo- and photo-therapeutics. Irradiation of the plasmonic Au nanostructures in the therapeutic window with 808 nm laser light results in a good photothermal response, which (i) is not affected by the presence of either the chemo- or the phototherapeutic guests and (ii) does not lead to their photoinduced decomposition. Besides, irradiation of the hybrid Au nanoassembly with the highly biocompatible green light results in the NO release from the NOPD with efficiency similar to that observed for the free guest. Preliminary biological experiments against Hep-G2 hepatocarcinoma cell lines are also reported.

## 1. Introduction

The past few decades have witnessed a tremendous attention on gold (Au) nanostructures because of their manifold applications in different fields spanning photonics, catalysis, sensing and medicine [1,2,3,4]. Among the unique properties of Au nanostructures, photothermia is one of the most intriguing in view of its biomedical applications [5]. It exploits a straightforward working mechanism based on the capability of the noble metal to absorb light in the visible (Vis)/near-infrared (NIR) regions because of its localized surface plasmon resonance (LSPR), and to convert the excitation light into heat with superb efficiency while exhibiting excellent photostability. This phenomenon is at the basis of the photothermal therapy (PTT), which represents one of the emerging unconventional treatment modalities for cancer and bacteria diseases with great prospects in the burgeoning field of nanomedicine [6]. The ease of manipulation of light in terms of intensity, wavelength, duration and location, combined with the nanodimensions of Au structures offer, in fact, the great advantage to confine a rapid increase of temperature in a very small volume with high spatiotemporal precision, inducing cellular death with great efficiency and selectivity [7,8,9].

In the frame of practical biomedical applications of photothermia, fabrication of Au nanostructures possessing LSPR falling in the therapeutic window (650–1300 nm) [10], where tissues, hemoglobin and water display high optical transmission, is highly desirable. Compared with spherical Au nano-objects, usually displaying LSPR around 530 nm, non-spherical Au nanomaterials such as nanorods, nanoshells, nanocrosses, nanostars, nanotriangles and nanoflowers, are much more suited for PTT applications since they all display LSPR in the NIR region [11,12,13,14,15,16]. However, the synthetic protocols involved in the preparation of these nanostructures require a range of components such as preformed seeds, non-benign reducing agents, sacrificial redox agents and surfactants, whose toxicity has been of concern [17,18]. Therefore, the development of environmentally sustainable procedures to obtain biocompatible NIR-active Au nano-objects is very challenging.

The three qualifying criteria in the preparation of metal-based nanostructures that should be evaluated from a green chemistry perspective have been outlined by Raveendran et al. in their seminal paper [19] and involve the use of (i) environmentally acceptable solvents, (ii) eco-friendly reducing agents, and (iii) benign particle-stabilizing capping species. Additionally, the use of non-conventional stabilizing strong ligands containing S, N or P is strongly encouraged to allow the further functionalization of the Au surface.

Extracts from the plants have been very successful in obtaining plasmonic Au nanostrctures by green procedures because of their widespread availability, low cost, environmental friendliness, and non-toxic nature of the large variety of natural reducing agents they contain [20,21,22,23,24,25,26,27,28,29,30]. We have also recently shown that water-dispersible, biocompatible NIR-absorbing Au nanostructures can be easily obtained in one step by exploiting as reducing agent the nitric oxide (NO) photogenerated through a NO photodonor (NOPD) covalently integrated within a β-cyclodextrin (β-CD) branched polymer [31]. In this case, the polymeric scaffold plays a double role as both templating and soft stabilizing agent for the noble metal.

**Scheme 1:**
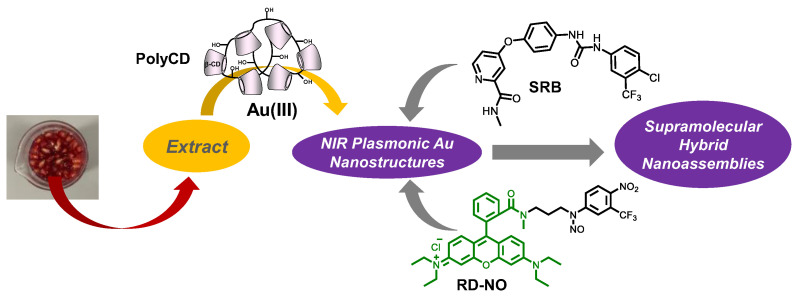
Schematic for the preparation of the NIR plasmonic Au nanostructures and their supramolecular assembling with SRB and RD-NO encouraged by the PolyCD polymer.

In this paper, we decided to merge these two approaches and report on the possibility to prepare NIR-active Au nanostructures by an eco-sustainable methodology, using *Punica granatum* (pomegranate) seeds extract (PSE) as a reductant in the presence of the branched polymer PolyCD (Scheme 1), which does not contain the NOPD. This polymer, consisting of β-CD units interconnected by epichlorohydrin spacers to form glyceryl cross-linked β-CD polymer, is highly soluble in water medium and highly biocompatible [32,33,34]. We also show that PolyCD can be exploited for the further supramolecular assembling of sorafenib (SRB) and the NOPD activatable with green light RD-NO (Scheme 1), chosen as representative for chemo- and photo-therapeutic components, to obtain supramolecular hybrid nanoconstructs for potential multimodal therapy.

## 2. Materials and Methods

### 2.1. Materials

All chemicals were purchased by Sigma-Aldrich and used as received. PolyCD was prepared by crosslinking β-CD with epichlorohydrin, under strong alkaline conditions, following a described method [35]. A mixture of different molecular weight compounds was obtained in the polycondensation reaction. Separation was performed with size exclusion chromatography (SEC) with water-compatible high-performance columns using pullulan standards. The β-CD content in PolyCD was ca. 70% *w*/*w*, as determined based on NMR spectra. RD-NO was synthesized according to our previously reported procedures [36]. SRB concentration was determined by absorption spectroscopy, using a molar extinction coefficient of 41,280 M^−1^ cm^−1^ at 265 nm in MeOH solution and 32,500 M^−1^ cm^−1^ at 268 nm when complexed within PolyCD. All solvents used for the spectrophotometric studies were at spectrophotometric grade. Ultrapure water (MilliQ) was used.

### 2.2. Instrumentation

UV-Vis absorption was recorded with a JascoV-560 spectrophotometer in air-equilibrated solutions, using quartz cells with a path length of 1 cm.

The size distribution was determined on a dynamic light scattering Horiba LS 550 apparatus equipped with a diode laser with a wavelength of 650 nm.

Transmission Electron Microscopy (TEM) grids were prepared by placing 400-mesh carbon-coated Cu grids on top of an aqueous dispersion of the sample for 10 min, plotting away the excess liquid, and air-drying the grid. Electron microscopy images were taken on a FEI Tecnai G2 operating at 100 kV, equipped with an Olympus Veleta camera. 

Photothermal experiments were performed by irradiating the samples (100 µL) in a NMR tube with a 808 nm continuous-wave (CW) laser (ca. 6 W cm^−2^) having a beam diameter of ca. 1.5 mm and detecting the temperature changes with a FLIR C3 thermal imaging camera. Pictures are edited using FLIR tools software and presented with a linear colour scale for temperature.

Absorption spectral changes were monitored by irradiating the sample in a thermostated quartz cell (1 cm path length, 3 mL capacity) under gentle stirring, using either 808 nm or 532 nm CW ca. (ca. 6 W cm^−2^) and having a beam diameter of ca. 1.5 mm.

### 2.3. Preparation of the PSE

Pomegranate fruits were collected in October from the pomegranate grove (Siracusa, Italy). The peels were separated from fleshy, juicy, sacs (arils) that surround the seeds.

To obtain PSE, 100 g of arils with seeds were added to 100 mL of a H_2_O:EtOH 50:50 (*v*:*v*) solution. After grinding, the solution was macerated for 2 h and filtered through a paper filter. Then, the filtered solution obtained was centrifugated (5000 rpm, 5 min) and the supernatant was collected. The solvent was removed under reduced pressure at 40 °C. The viscous, sticky product was precipitated with methanol (50 mL) in order to remove carbohydrates and proteins and the solid was filtered on a glass filter (porosity 4). The supernatant was recovered, and the solvent was removed under reduced pressure. The so-obtained PGE was used for Au nanostructures synthesis.

### 2.4. Synthesis and Characterization of the Au Nanostructures

PSE (4.5 mg mL^−1^) was solubilized with 3 mL of a water solution of PolyCD (2 mg mL^−1^). This solution was then added with 100 μL of a concentrated aqueous solution of HAuCl_4_ to achieve the final concentrations of 0.4 mM. The final mixture was left at room temperature under continuous stirring under air-equilibrated conditions and the spectral evolution was followed by UV-Vis spectroscopy as a function of time until no further spectral change was observed. The sample was centrifugated (14,000 rpm, 10 min), the supernatant removed and the precipitate was resuspended in water (3 mL) and characterized by UV-Vis absorption, DLS and TEM. The pH of the solution changed from 3.3 to 7.3 after centrifugating and resuspending the Au nanostructures.

### 2.5. Supramolecular Encapsulation of SRB and RD-NO in the PolyCD-Stabilized Au Nanostructures

Stock solutions of either SRB or RD-NO in MeOH were prepared and the solvent was evaporated under reduced pressure at 25 °C to form a thin film. The resulting film was rehydrated with 3 mL of a suspension of the Au nanostructures prepared as previously described and left overnight under continuous stirring at room temperature.

### 2.6. Biological Experiments

#### 2.6.1. Cell Lines

Hepatocellular carcinoma cell line (HepG2) was from American Type Culture Collection (ATCC, Manassas, VA, USA). Cells were cultured in Dulbecco’s modified Eagle’s medium with high glucose and without phenol-red (DMEM, Sigma-Aldrich, Milan, Italy) containing 10% fetal bovine serum (FBS), L-glutamine, 100 U mL^−1^ of penicillin and 100 μg mL^−1^ of streptomycin (all from Sigma-Aldrich, Milan, Italy) in a humidified incubator (Heracell 150, Thermo Scientific, Waltham, MA, USA) at 37 °C, 95% rel. humidity and 5% CO_2_.

#### 2.6.2. Cell Viability

Cells were detached from the bottom of flasks by trypsin-EDTA solution (Sigma-Aldrich, Milan, Italy), counted in a Bürker chamber, and plated into 96-well plates (EuroClone, Milan, Italy) at a density of 1×10^4^ for 48 h until the monolayer reached a confluency of approximately 80%. For in vitro evaluation, cells were exposed to the different samples alone for 4 h prior to irradiation with light. Cells were then washed, and the phenol red-free and FBS-free medium was added. The experimental plates were divided into two experimental groups: one was maintained in the dark, while the other was exposed to 808 nm, 532 nm or both CW laser light sources for 15 min. In each group, untreated cells were maintained as the control. After the irradiation, the FBS-free medium was removed and the complete medium added. All the plates were brought back to the incubator. After 24 h, the cellular metabolic activity was assessed by MTT assay. Briefly, after the indicated time, the medium was replaced with 180 μL of medium and 20 μL of MTT solution (stock solution at 5 mg mL^−1^) and the wells were incubated for 3 h at 37 °C. In this condition, mitochondrial dehydrogenases of viable cells conversed the tetrazolium ring of MTT into formazan crystals that were solubilized in 100 μL of DMSO. The absorbance was measured at 570 nm in a microplate reader (AMR-100, Allsheng, Hangzhou, China). The cytotoxicity index was calculated using the control cells as 100% viability. Cell viability after light exposure was calculated as percentage ± SD with respect to untreated cells kept in the dark.

## 3. Results and Discussion

**Figure 1:**
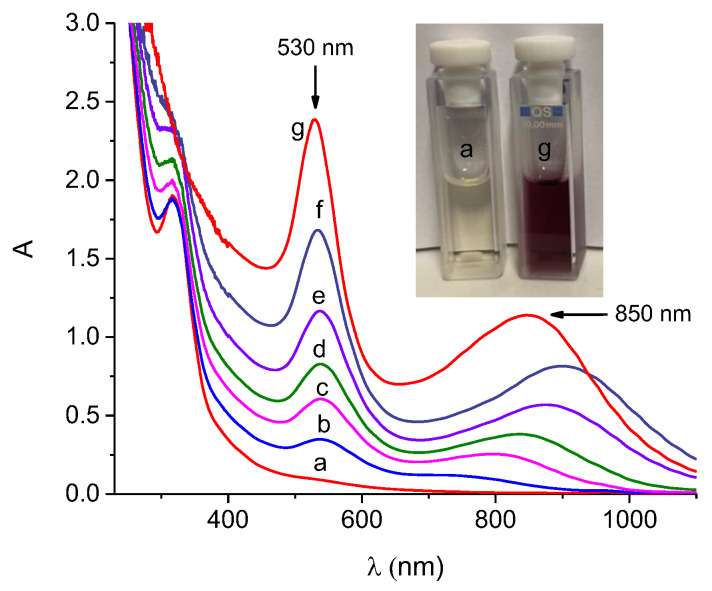
Absorption spectral evolution from 0 min (a) to 6 h (g) observed for an aqueous solution of PolyCD (2 mg mL^−1^) containing PSE (4.5 mg mL^−1^) in the presence of HAuCl_4_ (0.4 mM). The inset shows the actual image of the sample a and g, respectively. T = 25 °C.

Figure 1 shows the absorption spectral evolution observed as a function of the time for a solution of PSE in the presence of PolyCD and HAuCl_4_. We observed the formation of two LSPR absorption bands in the Vis and NIR regions at ca. 530 nm and ca 850 nm, respectively, typical for Au nanostructures and whose formation was complete in ca. 6 h. According to the known complexity of the process leading to the formation of Au(0) [37], no isosbestic points were observed during this kinetic phase.

**Figure 2:**
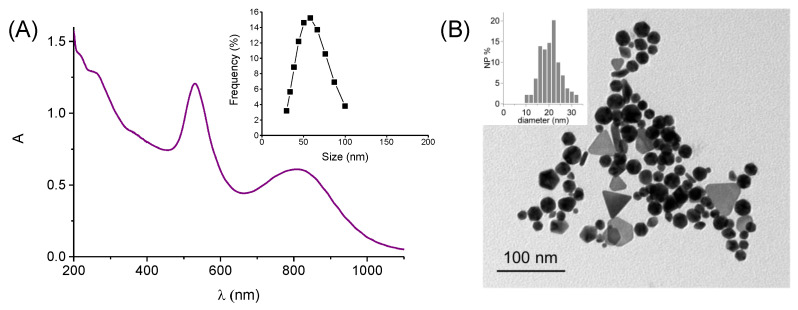
(**A**) Absorption spectrum of an aqueous dispersion of Au@PolyCD. The inset shows the size distribution of Au@PolyCD measured by DLS. (**B**) Representative TEM image of Au@PolyCD. The inset shows the size distribution of the almost-spherical NPs.

After centrifugation and removing the supernatant, the precipitate resulted as well dispersible in water (see experimental) and exhibited spectral features similar to those observed at the end of the reaction with both LSPR bands in the Vis and NIR regions at ca. 530 nm and 810 nm, respectively (Figure 2A). DLS measurements indicate the presence of Au nanostructures with a hydrodynamic diameter of about 55 nm (inset Figure 2A). TEM analysis (Figure 2B) revealed the formation of almost-spherical nanoparticles (NP) with an average diameter of 19 nm (inset Figure 2B) and non-spherical nanostructures, like triangles and pentagons, with a dimension between 36 and 45 nm. According to literature, the former are responsible for the LSPR localized in the Vis region, whereas the latter are for the LSPR localized in the NIR region [11,12,13,14,15,16,38]. The Au nano-objects remain dispersible and stable in water for weeks. This is reasonably due to the well-known effective capping capability of the β-CD units of the polymer to Au nanostructures, henceforth Au@PolyCD [31,39].

Note that the shape of the Au is strongly dependent on the ratio between the PSE and the Au precursor. In fact, only the LSPR centered in the Vis region, but not that in the NIR region, was observed for PSE concentrations higher than 4.5 mg mL^−1^ (i.e., 10, 20, 30 mg mL^−1^), while keeping constant the concentration of HAuCl_4_. This behavior was already observed in our previous work by changing the ratio between the reductant (NO radical) and the Au precursor [31], using the same polymeric scaffold of PolyCD and definitely deserves deeper investigation. Besides, control experiments performed in the absence of PolyCD showed the formation of the band in the Vis region within similar time intervals but only a weaker, non-defined, very broad absorption extending beyond 1000 nm with significant scattered light, indicative for the generation of poorly water-soluble aggregates (data not shown). This finding confirms that the presence of the polymeric network plays a key role in determining the shape, solubility and time stability of the Au nanostructures. We also believe that the formation of the observed plasmonic Au nanostructures under our experimental conditions can be encouraged by a redox process between PSE and Au(III), probably occurring within the polymeric scaffold, due to the well-known complexation capability of the β-CD branched polymer towards Au metal ions. This hypothesis is supported by the significant red-shift (ca. 20 nm) of the HAuCl_4_ absorption band observed in the presence of the PolyCD (λ_max_ ca. 310 nm) if compared to that in free water (λ_max_ ca. 290 nm), analogously to what was already observed for Au(III) in the presence of similar branched β-CD polymeric scaffolds [31,40].

**Figure 3:**
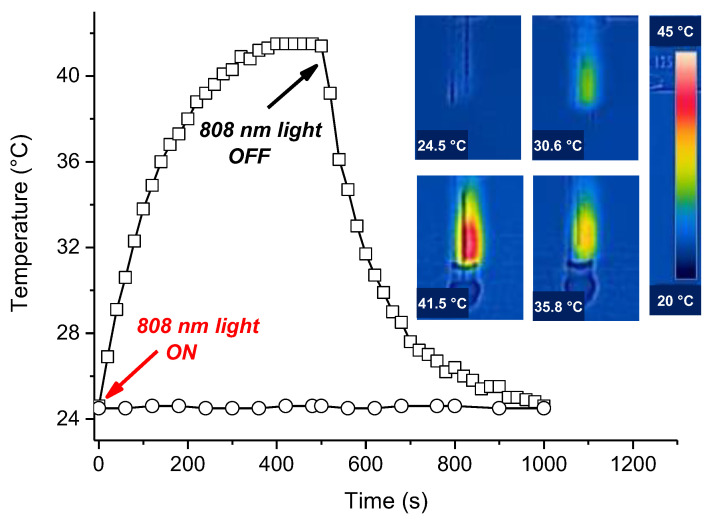
Temperature changes observed upon 808 nm CW laser irradiation (6 W cm^−2^) of the Au@PolyCD sample (100 µL) reported in Figure 2 (☐) and, for the sake of comparison, of pure water (◯). The inset shows representative thermographic images of the sample recorded after 0, 60, 140, and 400 s of irradiation (clockwise from top-left to bottom).

The potential use of Au@PolyCD as NIR-activatable photothermal agents was evaluated by irradiation with a CW laser at λ_exc_ = 808 nm and monitoring the temperature changes by using an infrared camera. Figure 3 shows a rapid temperature increase occurring in few minutes, reaching plateau values of ca. 42 °C (ΔT = ca. 18 °C) and decreasing as the laser light is turned off. In contrast, no significant changes were observed in the case of pure water upon otherwise identical irradiation conditions (see Figure 3).

Our choice to use the branched polymer PolyCD in this work was motivated not only to obtain NIR-active and stable Au nanostructures, but also to allow the achievement of supramolecular hybrid nanotherapeutics through a host-induced self-assembly [41,42] with additional therapeutic components. PolyCD represents, in fact, an intriguing scaffold for the supramolecular assembling of multiple guests with larger stability constants if compared with either the unmodified β-CD polymers or monomeric β-CD [33,34,35]. This is because of the possibility of interactions with different binding sites, such as the tridimensional network of the polymer, the hydrophobic cavities of β-CD or both. Furthermore, it has been extensively used in our group to integrate a variety of phototherapeutics and effectively deliver them into cancer cells [43,44,45,46].

As proof of concept, we decided to explore the capability of the Au@PolyCD to supramolecularly encapsulate the anticancer drug SRB and the NOPD RD-NO (see Scheme 1), chosen as representative examples of water-insoluble chemo- and photo-therapeutic guests.

SRB is a multi-kinase inhibitor, approved by the U.S. Food and Drug Administration, currently used in patients for the cure of hepatocellular and advanced renal cell carcinomas [47,48], and recently demonstrated to be an excellent guest for PolyCD-like hosts [49].

**Figure 4:**
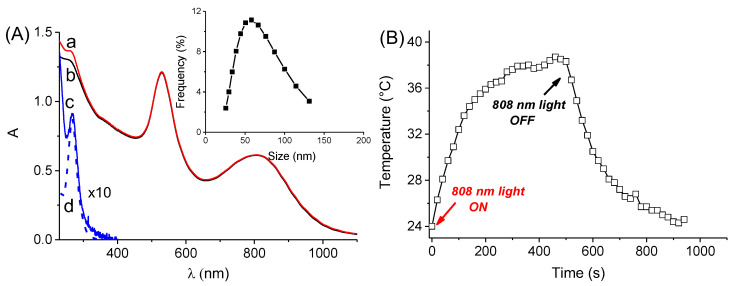
(**A**) Absorption spectrum of an aqueous dispersion of Au@PolyCD after (a) and before (b) stirring overnight with a thin film of SRB. Difference absorption spectrum between a and b (c) and absorption spectrum of a solution of PolyCD (2 mg mL^−1^) encapsulating SRB at the same concentration as in a (d); spectra c and d are multiplied for a factor of 10 for sake of clarity. The inset shows the size distribution of sample a measured by DLS. (**B**) Temperature changes observed upon 808 nm CW laser irradiation (6 W cm^−2^) of the Au@PolyCD@SRB complex (sample a).

Figure 4A (spectrum a) shows the absorption spectrum of a sample obtained after stirring overnight of thin films of SRB, obtained by drying a methanol solution of the drug, with a dispersion of the Au@PolyCD. The absorbance increases in the UV region at ca. 268 nm, typical for the SRB absorption, whereas it remains unaltered in the correspondence of the LSPR bands (see spectrum b in Figure 4A for comparison). This finding is indicative for the encapsulation of the chemo drug within the polymeric network as a result of the formation of the host–guest complex Au@PolyCD@SRB. Accordingly, the difference spectrum between the sample Au@PolyCD@SRB and Au@PolyCD (c in Figure 4A) results as very similar to that of SRB encapsulated within PolyCD in the absence of Au (d in Figure 4A). Based on the molar extinction coefficient of the PolyCD@SRB complex previously reported, a concentration of SRB of ca. 1.5 µg mL^−1^ can be estimated in the Au@PolyCD@SRB. Interestingly, the encapsulation of SRB leads to only a slight change in the size, which increased from ca. 50 to ca. 60 nm (inset Figure 4A) and does not modify the photothermal properties of the nanoassembly (Figure 4B), which were very similar to those observed in the absence of the chemo drug. Besides, the temperature increase does not induce any chemical/physical modification of the Au@PolyCD@SRB assembly as demonstrated by the unaltered absorption profile in the whole UV–Vis–NIR spectral range after the photothermal experiments (data not shown).

**Figure 5:**
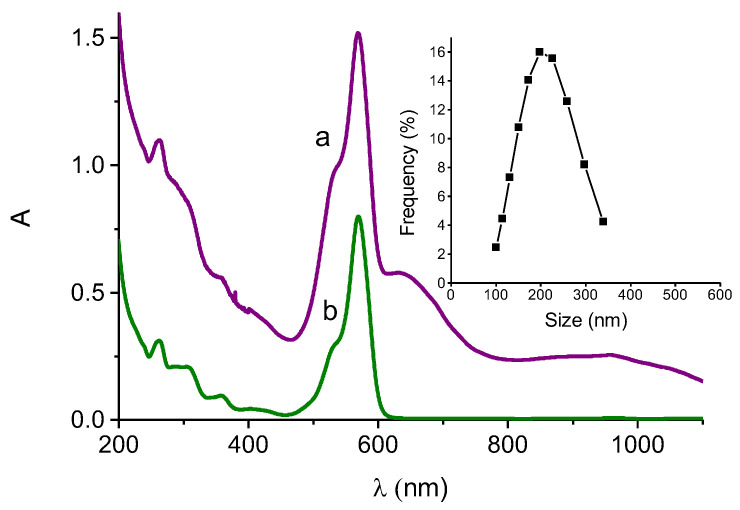
(A) Absorption spectrum of an aqueous dispersion of Au@PolyCD after stirring overnight with a thin film of RD-NO (a) and, for comparison, absorption spectrum of a solution of PolyCD (2 mg mL^−1^) encapsulating RD-NO (7 µM). The inset shows the size distribution of sample a measured by DLS.

RD-NO, an NOPD recently developed in our group [36], consists in a rhodamine antenna covalently linked to an N-nitroso appendage through a flexible spacer. Release of NO, a well-known anticancer species if produced within a specific concentration range, is triggered by an intramolecular electron transfer upon excitation of the antenna with the highly biocompatible green light and can be accurately regulated by tuning the excitation intensity [36]. RD-NO has proven to be an effective phototherapeutic against different cell lines and can effectively be encapsulated within PolyCD (see below). Analogously to SRB, RD-NO can also be entrapped by the Au@PolyCD, as demonstrated by the appearance of the typical absorption of the rhodamine chromophore (a in Figure 5) very similar to that observed for RD-NO incorporated within the PolyCD alone (b in Figure 5), as a result for the formation of the host–guest hybrid complex Au@PolyCD@RD-NO. However, in contrast to what was observed with SRB, encapsulation of RD-NO induces significant changes in the plasmonic spectral features of Au. In fact, both the green and NIR plasmon absorption bands shifted towards longer wavelength and decreased in intensity. This effect is not uncommon since it is typically observed for dyes adsorbed in close proximity of Au nanostructures when their spectra overlap strongly and is the result of a strong coupling between the molecular resonance of the dye (i.e., rhodamine) and the plasmonic resonance of Au [50]. The size of the Au@PolyCD@RD-NO resulted as ca. 4-fold bigger than that observed in the absence of RD-NO, being ca. 200 nm (inset Figure 5). This phenomenon is in good agreement with what was already reported for rhodamine-based dyes absorbed on Au nanostructures [51].

**Figure 6:**
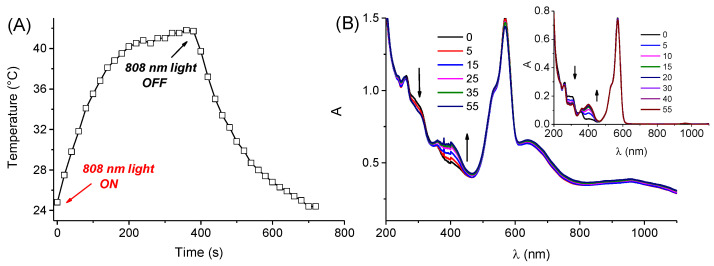
(**A**) Temperature changes observed upon 808 nm CW laser irradiation (6 W cm^−2^) for the aqueous dispersion of the Au@PolyCD@RD-NO complex. (**B**) Absorption spectral changes observed from 0 to 55 min of 532 nm CW laser irradiation (6 W cm^−2^) light irradiation of the aqueous dispersion of Au@PolyCD@RD-NO. The arrows indicate the course of the spectral profile with the illumination time. The inset shows the same spectral changes observed for the RD-NO (7 µM) encapsulated within PolyCD (2 mg mL^−1^) in the absence of Au.

The photoresponse of the PolyCD@Au@RD-NO complex was then evaluated in terms of photothermal and NO photoreleasing properties upon selective excitation with NIR and green light at 808 nm and 532 nm, respectively. Interestingly, despite the absorption in the NIR region being ca. 2-fold lower than that observed in the absence of RD-NO (see Figure 2 for comparison), PolyCD@Au@RD-NO exhibited a temperature jump profile (Figure 6A) very similar to that observed in the absence of RD-NO.

Also in this case, the temperature increase does not induce any chemical/physical modification of the Au@PolyCD@RD-NO, as demonstrated by negligible changes in the absorption profile after the photothermal experiments (data not shown).

Figure 6B shows the absorption spectral evolution observed upon irradiation of Au@PolyCD@RD-NO at 532 nm. Significant changes are observed only in the absorption region of RD-NO, whereas negligible modification is observed in correspondence of the Au LSPR bands. In particular, we observed the formation of a new absorption at ca. 400 nm and a bleaching at ca. 300 nm accompanied by the presence of fairly clear isosbestic points, indicative for a clean photochemical reaction. This photochemical profile is very similar to that previously observed for both the free RD-NO [36], in agreement with the light-triggered release of NO, and for RD-NO encapsulated within PolyCD in the absence of Au (inset Figure 6B).

**Figure 7:**
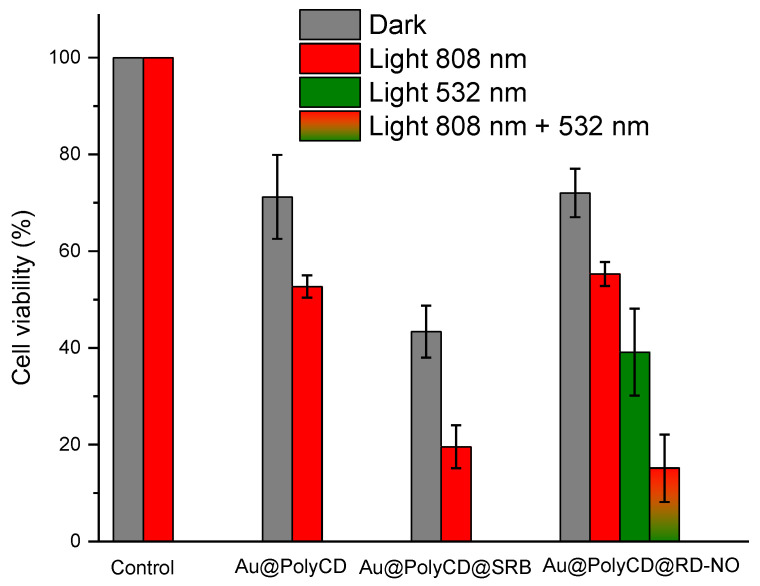
Cell viability observed 24 h after incubating HEP-G2 hepatocarcinoma cells with free aqueous dispersion of Au@PolyCD, Au@PolyCD@SRB and Au@PolyCD@RD-NO in the dark and upon different irradiation conditions (occurred after the first 4 h of incubation) for 15 min.

Au@PolyCD and the supramolecular complexes with SRB and RD-NO were stable in culture medium for more than one week, as demonstrated by the unchanged UV–-Vis–NIR absorption profile and intensity. This encouraged us to carry out very preliminary biological experiments to test the validity of the supramolecular hybrid nanoconstructs as potential multimodal therapeutics against Hep-G2 hepatocarcinoma cell lines and the results are illustrated in Figure 7. Au@PolyCD showed in the dark a moderate reduction of cell viability, which instead decreased upon irradiation with NIR light as a result of the photothermal effect induced by the noble metal. The dark cell viability further decreased in the case of the Au@PolyCD@SRB complex as a result of the anticancer action of SRB, despite the fact that it was used at the concentration of ca. 1.5 µg mL^−1^, which is below the IC_50_ value (3.8 µg mL^−1^) reported for the free drug for Hep-G2 cell lines [49]. Irradiation of this sample with NIR light leads to a significant reduction of cell viability, probably due to a chemo-photo combinatory effect.

Dark cell viability similar to Au@PolyCD was observed for Au@PolyCD@RD-NO. In this case, the light experiments were performed upon irradiation with 808 nm light, which trigger photothermia, 532 nm light, which trigger NO release, and with both irradiation sources. As illustrated in Figure 7, a significant reduction of cell viability was observed upon double irradiation, probably as a result of a synergism between the photothermal and the NO photodynamic action. Note that the shift of the green plasmon absorption of Au beyond 600 nm upon encapsulation of RD-NO in the PolyCD makes the NOPD the main absorbing species at 532 nm, ruling out that the decrease of cell viability using this excitation source can be attributable to an additional photothermal effect induced by Au nanostructures. This was confirmed by an increase in the temperature of only 5 °C upon irradiation with green light.

## 4. Conclusions

We have shown a simple eco-friendly procedure based on pomegranate extract as a reductant to obtain plasmonic Au nanostructures with LSPR in the NIR spectral region and stabilized by a highly biocompatible β-CD branched polymer. The spectral properties of these nanoconstructs allow their photoexcitation in the therapeutic window at 808 nm, resulting in a significant photothermia. Thanks to the excellent host capability of the branched polymer, the obtained nanoconstructs are able to encapsulate in a non-covalent fashion a conventional chemo-therapeutic such as SRB and an NOPD activatable with the biocompatible green light. This leads to intriguing supramolecular hybrid assembly conserving (i) the nanodimensional character, (ii) the photothermal activity of the Au component, (iii) the anticancer action of the chemo-therapeutic and (iv) the NO photorelease properties of the NOPD. The suitability of these nanoassemblies in the prospect of multimodal anticancer applications has been demonstrated by preliminary experiments against Hep-G2 hepatocarcinoma cell lines, which revealed synergistic action between the cytotoxic species involved.

## Data Availability

Not applicable.
